# Supplementary material for: Saturation genome editing of BAP1 functionally classifies somatic and germline variants
Source: Nat Genet. 2024 Jul 5;56(7):1434–45. doi: 10.1038/s41588-024-01799-3 (PMC11250367; doi:10.1038/s41588-024-01799-3)
Supplement: Supplementary file 1 — Supplementary Notes 1 and 2, Methods 1−14 and Fig. 1. [file 41588_2024_1799_MOESM1_ESM.pdf]

nature genetics

Supplementary information https://doi.org/10.1038/s41588-024-01799-3

Saturation genome editing of *BAP1* functionally classifies somatic and germline variants

In the format provided by the authors and unedited

Supplementary Notes:

Supplementary Note 1: Assessment of *BAP1* variants in cancer and neurodevelopment

Nine *de novo* missense *BAP1* variants (2 recurrently observed) have been associated with Küry-Isidor Syndrome[9]. In addition, a further 7 *de novo* heterozygous variants (4 missense, 2 frameshift and 1 synonymous) were identified by a recent meta-analysis of over 31,000 developmental disorder families[24]. Of these 16 variants, 15 are assayed in our screen (1 frameshift is not present). 13/15 reported single nucleotide variants are depleted by SGE; 4/15 weakly depleted and 9/15 strongly depleted (Extended Data Fig.7i). Küry *et al.*[9] performed cDNA-based rescue experiments assaying the abundance of the BAP1 substrate H2AK119ub in HAP1 cells. Variants assayed by Küry *et al.* are shown in Fig.4h. Out of 6 variants tested c.34C>A (P12T), c.271T>C (C91R), c.272G>C (C91S) and c.506A>G (H169R) were unable to restore H2AK119ub to wild-type levels; all are strongly depleted by SGE. c.34C>G (P12A) was shown by Küry *et al.* to partially restore H2AK119ub levels, suggesting partial enzymatic activity on this substrate, and consistent with this we find this variant to be weakly depleted, suggesting it may be a hypomorphic allele. However, it is not clear if this molecular phenotype translates to a less severe clinical phenotype[9]. c.2153G>A (R718Q) was found to restore H2AK119ub to wild-type levels, and is classified as unchanged by SGE (Fig.4h).

In addition to the 6 development-associated variants tested, a further 20 have been characterised using a BAP1/ubiquitin assay in H226 cells[25]. 17/20 variants were from germline sequencing of melanoma cases, while 3/20 were from controls. 19/20 variants tested, all missense, were present in our screen (an insertion, '3:52437191,-/A', which was not tolerated in the ubiquitin assay, was not present). S98R showed compromised ubiquitinase activity in H226 and is strongly depleted by SGE (Fig.4h). 18/20 variants tested were found to have uncompromised ubiquitinase activity, 17 of which are classified as unchanged by SGE (Fig.4h). Intriguingly, the variant c.1217A>T (gAg>gTg, E406V), found in a melanoma patient, had wild-type levels of deubiquitinase activity, and is classified as enriched by SGE (Fig.4h). An alternative allele leading to the same missense change, c.1217_1218delinsTC (gAG>gTC, E406V) is classified as unchanged. As c.1217A>T did not deplete and has a spliceAI[42] score of 0.4, higher than c.1217_1218delinsTC (0.17), it is possible that c.1217A>T compromises *BAP1* through a mechanism other than a loss of deubiquitinase activity.

Germline *BAP1* variants identified in *BAP1*-Tumour Predisposition Syndrome (TPDS) families are known to be associated with a wide variety of cancers[26]. We analysed data from a comprehensive clinical analysis of 181 families believed to have TPDS. These families carried 140 unique variants across 1,392 individuals, of which 653 were confirmed to have variant carrier status. 85/140 unique variants are observed in our screen (including 34/36 missense, see Supplementary Table 5). 27/85 are weakly depleted and 33/85 are strongly depleted (25/85 unchanged). We see a significantly earlier age of onset for depleted variant carriers compared to unchanged variant carriers ($p<0.01$, two-sided Mann-Whitney-Wilcoxon Test), as has previously been seen between null and missense variant carriers, but we see no difference between strongly/weakly depleted classifications in this patient group[26] (Extended Data Fig.7j). We observe that strongly and weakly depleted variants are distributed between different cancer types for individuals with confirmed carrier status (Extended Data Fig.7k). Extending this analysis to somatic variants; 195/268 unique *BAP1* variants identified in MSK-

IMPACT are found in our screen with 70/195 strongly depleted and 74/195 weakly depleted (48/195 are unchanged and 3/195 are enriched) across a variety of cancer types (Extended Data Fig.7k)[43].

Supplementary Note 2: *BAP1* disruption associates with cancer and high IGF-1 in UK

Binomial and Gaussian regression models were used to associate UKBB traits with SGE-depleted variants (see Supplementary Method 14). SGE-depleted non-synonymous variants were found to be significantly associated with all-site cancer pre-disposition. An analogous mask, created without SGE data, composed of missense variants predicted to be disruptive by CADD[21] combined with HC PTVs was not significantly associated with cancer (*p*=0.108, n=643), demonstrating the relatively higher specificity of SGE compared to CADD predictions for these alleles. In addition to CADD, we also assessed EVE[16] and REVEL[56] masks across cancer phenotypes, finding no significant association with a cancer diagnosis (Fig.5a & Extended Data Fig.8a). Of note, the percentage of patients with a cancer diagnosis is higher in SGE-depleted non-synonymous variant carriers than non-carriers (Supplementary Table 7), however not all SGE-depleted masks have a significant effect, for example SGE-depleted missense variants in all cancers combined (*p*=0.243, n=43) (Fig.5a, Extended Data Fig.8a, Supplementary Table 7). This is likely due to low power and PheWAS effect size of some variants, with few (n=43) missense *BAP1* carriers observed in UKBB, and the observation that SGE-depleted high-confidence (HC) protein truncating variants (PTVs) have roughly double the effect compared with SGE-depleted missense variants (0.898 and 0.411, respectively), when all cancers combined are assessed. Consistent with this, SGE-depleted missense variants significantly associate with solid cancers (i.e., excluding blood), with blood cancers generally not linked to *BAP1* mutation/loss[67] (Extended Data Fig.8a). Importantly, we find that the average age of cancer onset for SGE-depleted non-synonymous *BAP1* variant carriers and non-carriers in UKBB is similar at 62.54 (n=24) and 60.71 (n=95,185) years, respectively (60.57 years, n=9,071, for SGE-unchanged *BAP1* variant carriers).

We analysed the association between SGE-depleted variants and quantitative traits other than cancer, identifying significantly higher IGF-1 in SGE-depleted non-synonymous variant carriers compared to non-carriers. Generalized linear model regression analysis gave a *p*-value of 1.17e-03 for SGE-depleted non-synonymous carriers and a mean IGF-1 level of 23.58 nmol/L (n=69). Non-carriers have a mean IGF-1 level of 21.35 nmol/L (n=398,505). When all *BAP1* HC PTVs in UKBB are added to SGE-depleted non-synonymous variants, an increase in effect significance is not seen (*p*=9.77e-04, IGF-1 level=23.36 nmol/L, carriers n=79) demonstrating that the association with increased IGF-1 level is robust (Extended Data Fig.8b). Importantly, we do not see a difference in mean IGF-1 levels between SGE-depleted non-synonymous *BAP1* variant carriers with cancer (IGF-1 level=22.19 nmol/L), and those without cancer (IGF-1 level=24.36 nmol/L, *p*=0.19). Likewise, non-carriers with and without cancer have similar mean IGF-1 levels, 20.85 and 21.51 nmol/L, respectively.

Supplementary Methods:

Supplementary Method 1: Essentiality phenotyping – cell counting

In order to confirm essentiality of *BAP1* in HAP1 cells, sgRNAs targeting *BAP1* and a known non-essential gene, *HPRT1,* were transfected in 6 well plates seeded with $1x10^5$ Polyclonal Cas9+ *LIG4*- cells with 2µg sgRNA plasmid and 0.6µL Xfect (Takara) in 50µL with Xfect buffer (transfection conditions as in Methods: 'Tissue culture, cell transfection and sampling'). Pre-cloned sgRNAs (in backbone 'pKLV-PB-U6gRNA(BbsI)-PGKpuro2ABFP', Addgene, 50946) previously reported[68] were used. In Extended Data Fig.2a, *BAP1* gRNA-A: GCATGAGTTGCACAAGAGTT and gRNA-B: TTGAAGCGGATGTCGTGGTA and *HPRT1*: gRNA-A: TATTCCTCATGGACTAATTA and gRNA-B: TCTTGCTCGAGATGTGATGA were used. Empty backbone was also used as a control. The transfected cells were selected with puromycin at 3µg/mL for 3 days, followed by passaging and cell counting with trypan blue stain (Gibco) on a Countess machine (Thermofisher). Cell counting, washing and passaging were performed on Day 0, 1, 5, 7 and 9 – 100% cells were passaged, into 6 well plates. Multiple wells were used per sample for culturing when over-confluent, with pooling at timepoints.

Supplementary Method 2: Essentiality phenotyping – Indel analysis

An sgRNA targeting exon 5 of *BAP1* was transfected and selected as described in Methods: 'Tissue culture, cell transfection and sampling'. The polyclonal Cas9+ *LIG4*- line was used for this assay. HDR template library was not co-transfected, resulting in cells likely being repaired through a non-HDR pathway, creating indels. Edited cells were harvested on Day 5 and Day 11, and then processed for sequencing as described in Supplementary Methods 8-12. gDNA libraries were sequenced on the Illumina Miseq platform using a 600-cycle V3 kit for a 300 PE run. For indel analysis, sequencing counts or indels were ranked based on sequence abundance and mutational consequence (frameshift or inframe deletion) achieved through the following informatic processes:

Indel frequencies were generated using the 'indel' quantification mode nf-sge version 0.0.1 (https://github.com/team113sanger/Waters_BAP1_SGE/tree/develop/pilot_SGE_software/nf-sge__version-0.0.1); a Nextflow[58] pipeline and prototype version of QUANTS (https://github.com/cancerit/QUANTS). Within nf-sge, adapter trimming was performed with Cutadapt version cutadapt 2.5[60] with Python 3.6.8 (*--cores 1 --error-rate 0.5 --times 1 --overlap 3 -g file:[R1 adapters] fasta --pair-filter any -G [R2 adapters]*). EMBOSS[69] Needle[70] version 6.6.0 was used to perform a global alignment of the trimmed reads (*-auto -asequence [library] -bsequence [fastq] -sformat2 fastq -aformat3 sam -aextension3 sam -gapopen 10 -gapextend 0.5 -endopen 10 -endextend 0.5*). The SAM-formatted alignments were then parsed with Awk to determine the frequency of each unique CIGAR string (*awk -F"\t" '{print \$6}' ${sam} | sort | uniq -c | sort -nr*).

Supplementary Method 3: Essentiality phenotyping – targeted CRISPR/Cas9 screen

HAP1-A5 cells were transduced with an sgRNA lentiviral library containing 8,152 sgRNAs. The library was composed of: sgRNAs within all exons of 30 key genes of interest (including *BAP1*), within select exons of 977 other genes of interest, and 100 non-essential control genes.

Previously reported non-targeting guides were also included as a control for genotoxic stress[71,72]. Oligonucleotide sequences were ordered (Sigma) annealed and cloned into 'pKLV2_U6gRNA5(BbsI)_ccdb_PGKpuro2ABFP_W' (Addgene, 67974). Lentiviral particles were then prepared, and titrations were performed to discern the concentration necessary for a low multiplicity of infection (MOI) at ~1 sgRNA/cell. Lentivirus was added to T225 flasks containing sufficient HAP1-A5 cells to give 300X coverage, followed by overnight incubation. FACS on vector transduced cells was performed to confirm transduction efficiency at 30% (approximated MOI=0.36, by Poisson's distribution estimation). Puromycin (InvivoGen) was added to media at 0.7µg/mL for selection. Cells were passaged every 2-3 days with a 1:3 splitting ratio. Three cell pellets (containing $1x10^8$ cells each) were collected, washed and frozen for each timepoint (Day 7, 10, 14, 19, 23 and 28). 9µg of extracted gDNA (DNeasy Blood and Tissue kit, Qiagen) was split over x3 50µL PCR reactions (Q5® High-Fidelity DNA Polymerase 2X Master Mix, NEB), for first-round PCR (61°C annealing, 25 cycles) using primers (gLibrary-HiSeq_50bp-SE-U1: ACACTCTTTCCCTACACGACGCTCTTCCGATCTCTTGTGGAAAGGACGAAACA and gLibrary-HiSeq_50bp-SE-L1: TCGGCATTCCTGCTGAACCGCTCTTCCGATCTCTAAAGCGCATGCTCCAGAC). 10ng of plasmid library was also sampled to assess sgRNA composition/representation. PCR samples were purified using a DNA clean and concentrator kit (Zymo Research), eluting DNA in 50µL of EB buffer. Second round PCR (KAPA HiFi HotStart ReadyMix, Roche) to add indexing sequences was performed, and was purified with Ampure XP bead (Beckmann-Coulter) clean-up at a 0.7x ratio (as described in Supplementary Methods 8-12). Libraries were diluted to 4nM with 30% PhiX (Ilumina) spike-in and sequenced with 19bp SE reads using the custom sequencing primer, U6-Illumina-seq: TCTTCCGATCTCTTGTGGAAAGGACGAAACACCG. Sequencing files were processed through the MAGeCK[73] pipeline to obtain counts and Log_2-fold change (LFC) values.

Supplementary Method 4: Essentiality phenotyping – pilot SGE

A minimal SGE screen was performed using exon 5 sgRNA-A and a 496 variant HDR template library created through bespoke Python scripts[17]. Synthesis and cloning of the sgRNA and variant library were performed as described in Supplementary Method 7. Transfection was performed as in Methods: 'Tissue culture, cell transfection and sampling', except that polyclonal Cas9+ HAP1 *LIG4*- cells were used. Cells were sampled on Day 5 and Day 11, as has previously been reported[10] (rather than Day 4, 7, 10, 14 and 21 used in the main SGE experiment). Samples were then processed as in Supplementary Methods 8-12. gDNA and plasmid libraries were sequenced as separate pools with a 300 PE run on Illumina Miseq platform using a 600-cycle V3 kit. Informatic processes are as follows:

HDR library frequencies were generated using 'nf-sge' version 0.0.1 (https://github.com/team113sanger/Waters_BAP1_SGE/tree/develop/pilot_SGE_software/nf-sge__version-0.0.1); a Nextflow[58] pipeline and prototype version of QUANTS (https://github.com/cancerit/QUANTS). Nextflow version 19.10.0 was used to run nf-sge with an underlying Docker container providing the software dependencies. Adapters were removed from the raw sequencing data using Cutadapt version 2.5[60] with Python 3.6.8 *(--error-rate 0.5 --times 1 --overlap 3 --pair-adapters --pair-filter any -g [R1 adapter] -G [R2 adapter])*. A bespoke Perl script (library_quantification.pl) was used to determine the frequency of each HDR template using exact string matching to the reads from the trimmed FASTQ. Indel frequencies were

calculated with a bespoke R script (aln2cigar.R) to parse allele frequencies generated by CRISPRESSO2[74] version 2.0.34. The allele frequency table contains reference and alternative sequences, which are aligned and used to generate a representative CIGAR string. Indel frequency was calculated as the sum of each unique CIGAR sequence. The repository contains nf-sge , instructions for building the Docker container and example commands for nf-sge , CRISPRESSO2 and the bespoke R script for indel quantification: https://github.com/team113sanger/Waters_BAP1_SGE/tree/develop/pilot_SGE_software

Supplementary Method 5: HDR library generation – Design of variant libraries

VaLiAnT[13] version 1.0.0 was used to generate annotated variant sequence libraries based on the GRCh38 reference genome. Target regions were defined by primers, generated through Primer3 in Geneious™. Regions were designed to be ~245bp, to include an appropriate sgRNA target site, to span exon CDS and to include exon-flanking non-coding sequence. For *in silico* variant sequence generation, each target region was divided into three ranges: r1, r2 and r3 (see VaLiAnT wiki for generic design principles https://github.com/cancerit/VaLiAnT/wiki/). r2 is the exon CDS and r1 and r3 flanking intronic sequence for exons; 1-10, 14-16. For larger exons (11-13 and 17) r2 is a section of the CDS (with multiple partially overlapping target regions designed to cover the whole exon CDS) and flanking non-coding r1 and r3 sequence included where appropriate. Any sequence in the target region not defined by r1-3 ranges is considered constant and are unchanged from GRCh38. All regions included 1bp deletions and all possible SNVs, using the '1del' and 'snv' functions, respectively. In addition, CDS regions included an alanine scan ('ala'), stop scan ('stop') and codon deletion scan ('inframe'). The mutator function 'snvre' was also used in CDS sequence, which produces an alternative triplet codon sequence for each missense change generated by the 'snv' function and generates all synonymous mutations at each codon position. This function is helpful to increase the number of likely-unchanged variants within smaller exons for normalisation. r1 and r3 sequences were between 25-90bp and included 1bp deletions, all SNVs, and tandem base-pair deletions (using '2del0/1' function) to remove splice donor/acceptor sequences. All sequences generated included appropriate PAM/protospacer protection edits (PPEs) introduced through the software to create two synonymous mutations at defined sgRNA target sequences, in order to prevent unwanted re-cutting of incorporated HDR tracts by the sgRNA-Cas9 complex. Variants falling within target regions present in gnomAD (version 3) and ClinVar (release downloaded 2020-09-27) were included in variant libraries. Exon boundary coordinates were derived by VaLiAnT using a GTF for the MANE *BAP1* transcript ENST00000460680.6 filtered from the GENCODE basic annotation set (version 40). VaLiAnT was run separately to produce two libraries for each target region, Library A and B, with a different set of PPEs between the two libraries. Ilumina P5 (AATGATACGGCGACCACCGA) and P7 (TCGTATGCCGTCTTCTGCTTG) adapter sequences were appended to all oligonucleotides in order to perform a generic amplification of all designed target regions to increase starting material for downstream cloning processes. Any variant produced by the incorporation of ClinVar or gnomAD insertions that resulted in oligonucleotides >300bp were excluded. All unique variant sequences for each target region were combined to produce two oligonucleotide pools (Library A and B), generated by Twist Bioscience.

Supplementary Method 6: HDR library generation – Cloning of wild-type homology arms

In order to clone wild-type homology regions to flank variant containing CDS, primers were designed to amplify ~750-1000bp either side of exons using the Primer3 tool within Geneious™. Cloning adapters for NEBuilder® HiFi DNA Assembly (Master Mix, NEB) with cloning vector sequence were added to the 5' of the primer sequence (oligo suffix: hdr_f and hdr_r, Supplementary Table 9). Genomic DNA was extracted from the HAP1-A5 cell line using columns with RNAseI and Proteinase K incubations (Qiagen DNeasy Blood and Tissue kit). PCR was performed using KAPA HiFi HotStart ReadyMix, using 'hdr' oligos and 500ng of HAP1 gDNA in a 50µL reaction, with <25 cycles at an appropriate annealing temperature (generally 65°C) and 1.5-minute extension at 72°C. Amplicons were resolved on 0.8% agarose-TAE gels to check for single bands of the correct size and gel extraction (Qiagen QIAquick Gel Extraction Kit) was performed. A plasmid vector fragment comprising cloning adapter sequences, an origin of replication and ampicillin resistance marker was amplified by PCR (KAPA HiFi HotStart ReadyMix, 63°C annealing, <25 cycles) with 100ng of 'pMin-U6-ccdb-hPGK-puro' as template and 'hr_frag_f/r' primers (Supplementary Table 9), followed by DpnI digestion and gel extraction (Qiagen QIAquick Gel Extraction Kit) of the desired 1.8kb band resolved on a 0.8% agarose-TAE gel. Wild-type amplicons were incubated with the vector fragment at a 2:1 molar ratio (50ng of vector fragment was used) together with NEBuilder® HiFi DNA Assembly Master Mix (NEB) at 50°C for 1hr in a PCR machine. A 1:4 dilution with water was performed and 2µL was transformed into 50µL TOP10 (Invitrogen) competent cells and selected overnight on ampicillin agar plates (100µg/mL) at 37°C. Colonies were selected, grown in liquid culture and miniprepped (QIAprep Spin Miniprep). Wild-type sequence clones were confirmed through Sanger sequencing (Eurofins) using 'guide_seq_f/r' primers (Supplementary Table 9). Wild-type plasmid clones were used as templates in separate PCR reactions to linearize the homology arm and vector backbone regions and to exclude the wild-type exon CDS (and flanking non-coding sequence). 10pg of wild-type plasmid clone was used as template in 50uL PCR reactions (KAPA HiFi HotStart ReadyMix, annealing temperature 55-60°C for 35 cycles with 2.5min extension step). After PCR, reactions were treated with DpnI (NEB) to digest methylated plasmid template (37°C 2hrs, followed by 80°C 20min to inactivate the enzyme). Samples were then resolved on 0.8% agarose-TAE gels and gel extractions performed (Qiagen QIAquick Gel Extraction Kit). The purified linearized homology arm amplicons were then assessed by nanodrop for purity and concentration (samples were ~50ng/µL).

Supplementary Method 7: HDR library generation – Cloning variant libraries

In order to maintain library complexity and avoid over/under amplification, all library amplification PCR reactions were pre-optimized based on trial reactions run on a qPCR machine, using EvaGreen (Biotium) and ROX (Roche) dyes in 50µL reactions. Amplification curves were used to determine ideal cycle numbers and annealing temperatures (reactions were run at 60°C, 63°C and 65°C). Melt-curve analysis and agarose gel electrophoresis were used to confirm single band amplicons. Reactions were then re-run without dyes with optimal conditions on PCR machines for downstream cloning as follows: Oligonucleotide pools received from Twist contained 352.1ng and 361.5ng for Library A and Library B, respectively. Lyophilized DNA was resuspended in 10mM Tris-HCl pH 8 to give 10ng/µL stocks. To maintain library complexity 5 separate 50µL PCR reactions (KAPA HiFi HotStart ReadyMix, 60°C

annealing, 12 cycles) were performed for each library containing 20ng of oligo pool template in each reaction, and a final concentration of 0.3µM P5 and P7 primers. After PCR cycling, 5µL of Exonuclease I and Exonuclease I buffer (NEB) was added to each reaction and incubated at 37°C for 15mins, then 80°C at 15mins to remove single-stranded DNA library template and primers. Reactions were purified using MinElute columns (Qiagen), eluted in 10µL EB buffer. The replicate reactions pooled together after purification. Purified amplicon pools were run on 2% agarose-TAE gel and single band ~300bp confirmed. The samples were then quantified by nanodrop and 40ng of P5-P7 amplified the amplified pools were then used as template in PCR reactions (KAPA HiFi HotStart ReadyMix, annealing 63-65°C, 13-15 cycles). The specific target regions were amplified using 'lib_f/r' suffixed primers at a final concentration of 0.3µM (Supplementary Table 9). Reactions were performed in duplicate for each target region (20ng template in each reaction). After PCR cycles, reactions were treated with Exonuclease I and purified as above. Target region specific amplicons (50ng) were combined with the corresponding linearized homology arm amplicons (50ng) in 20µL reactions with NEBuilder® HiFi DNA Assembly Master Mix and incubated at 50°C for 1hr. 2µL of the assembly reaction was transformed into 100µL high-efficiency, chemically competent Stellar *E.coli* cells (Takara). After transformation, 1% of the cell suspension was plated on ampicillin agar plates (100µg/mL) to estimate transformation efficiency, the remaining 99% was cultured in 125mL LB with ampicillin in conical flasks at 37°C shaking (200rpm) overnight. Transformations with an estimated <50X coverage (less than 50 colonies per variant in the library) were repeated with optimization. 500µL was used to make glycerol stocks which were banked at -80°C. The remaining culture was used for maxipreps (Qiagen) in which culture was split over three 50mL tubes (Falcon), centrifuged for 10mins at 6000g, then pooled into 8mL suspension of P1 buffer and processed using 'high yield' Qiagen protocol, with elution in 200uL of EB buffer. Plasmid purity and concentration were assessed by nanodrop and stored at -20°C, those with concentrations <~1µg/µL were repeated with optimization from glycerol stocks. Plasmid library composition was assessed to confirm variant representation on the Illumina MiSeq platform, using 300 PE reads and processed with the QUANTS pipeline (see Supplementary Methods 10-12 for sequencing library preparation).

Supplementary Method 8: gDNA extraction and sequencing – gDNA extraction

gDNA was extracted from cell pellets using the Qiagen DNeasy Blood and Tissue kit, following the spin column protocol for cultured cell samples, with RNAse A (Qiagen) treatment for 15 min at 37°C before lysis. Samples were eluted in 100µL AE buffer and quantified and purity-checked by nanodrop. Samples with concentration higher than 50ng/µL and with A260/A280 values of 1.8-1.9 and A260/A230 values >2 were used for subsequent PCR steps. Repeat extractions were performed if the purified gDNA did not meet these criteria.

Supplementary Method 9: gDNA extraction and sequencing – primary PCR

Three PCR steps were performed for sequencing library preparation, all using KAPA HiFi HotStart ReadyMix polymerase (Roche) in 50µl reactions with 0.3µM final concentration of each primer. The primary reaction was to enrich for the edited loci, the secondary PCR to add Illumina sequencing adapters for indexing, the indexing PCR reaction to index sample-replicates for pooling and demultiplexing. For the primary PCR, a total of 3µg of gDNA (which equates to ~$1x10^6$ haploid genomes), split over three reactions containing 1µg gDNA was used

to maintain complexity and avoid 'jackpotting' bias. One primer in this reaction was designed to be outside of the cloned homology arm region to avoid amplifying any remaining HDR plasmid library in the sample ('gdamp_f/r' suffixed primers, Supplementary Table 9), giving amplicons of ~1.2-1.9kb. Reactions were pre-optimized as described above with EvaGreen (Biotium) on a qPCR machine to determine ideal cycle numbers and annealing temperatures, sampling reactions were then re-run without dyes. Most reactions had an optimal annealing temperature of 63-65°C and an optimal cycle number of 20-25. Triplicate PCRs were then pooled and purified through QIAquick columns (Qiagen, with 3µL NaOAC added to PB buffer to adjust pH) and eluted in 40µL EB buffer. To remove any carry-over of primer, which could interfere the secondary PCR, the purified products were then treated with 5µL Exonuclease I (NEB), with 10µL Exonuclease I buffer in a total volume 100µL with water, followed by incubation at 37°C for 20 mins then 80°C 20 mins. The reaction was then purified using a MinElute column (Qiagen), eluted in 15µL EB buffer and quantified by nanodrop. 10ng/µL dilutions of the products were made, which were then used as template for the secondary PCR.

Supplementary Method 10: gDNA extraction and sequencing – secondary PCR

Oligos with 'seq/r' suffixes (Supplementary Table 9) were used to append adapters to the edited target region sampled in the primary PCR amplicons, to give amplicons of ~400bp. Secondary PCRs were performed with optimal annealing temperatures of 55-66°C and an optimal cycle number of 13-18 cycles. 75ng of primary amplicon was used as template over 3 reactions, which were pooled and purified as described in the purification of primary PCR. Concentration and purity were assessed by nanodrop. Secondary PCRs with a yield less than 100ng were repeated.

Supplementary Method 11: gDNA extraction and sequencing – plasmid library PCR

HDR plasmid libraries were assessed for composition. 100ng of plasmid was used as template, split across four 50µL reactions for each library, with 'seq/r' oligos used. Annealing temperatures and cycling conditions were the same as used for the primary PCR amplification. Products were purified as described in the 'primary PCR' step. Products were diluted to 5ng/µL for use as template in indexing PCRs.

Supplementary Method 12: gDNA extraction and sequencing – indexing PCR and sequencing

Indexing PCRs were performed to give distinct indexes for each sample/timepoint/replicate submitted in a sequencing run. 25ng of secondary PCR was used as template in a single indexing PCR reaction, annealing was performed at 60°C for 7 cycles, using 'D501/2/3/4' i5 index and 'iPCRtag' i7 oligos (Illumina, Supplementary Table 9). Indexing PCRs were purified using Ampure XP beads (Beckmann-Coulter) according to the manufacturer's protocol with a 0.9X bead ratio. The purified product was eluted in 33µL EB buffer and was quantified by nanodrop followed by normalization to 10ng/µL. 15µL of concentration-normalized libraries were prepared, 8µL was run on a 2% agarose-TAE gel at 120V for 45mins to check for a single band at ~450bp with equal intensity seen across samples. Then, 5µL of each normalized library was pooled. All libraries for the same target region were processed, pooled and

sequenced together. Pooled sequencing libraries at 10ng/µL were quantified using a qPCR machine (KAPA Library Quantification Kit) and assessed by TapeStation (Agilent), diluted to 8pM and run on Illumina platform, with 20% PhiX (Illumina) spike-in. gDNA libraries were run on an Illumina Rapid HiSeq2500 v2 500 cycle platform with SE reads. gDNA libraries were split over 6 HiSeq2500 rapid runs. Plasmid libraries were run on an Illumina Miseq V3 600 cycle (300 PE) platform.

Supplementary Method 13: Pathogenic and benign truth sets for ACMG evaluation

In the construction of the pathogenicity truth set for evaluation of assay performance, we used all variants covered by our assay with a VEP functional consequence of frameshift or stop-gained, excluding those located in the final exon (exon 17). Our approach for benignity truth set construction first required establishment of a maximum tolerated allele frequency (MTAF) for *BAP1*, as described by Whiffin *et al.*[75]. We used the following parameters reflecting epidemiological features of uveal melanoma (UM), a canonical phenotype associated with pathogenic variants in *BAP1*:

- Disease prevalence: 1 in 1,290
    - This is based on a reported frequency of eye cancer of ~1 in 900, of which approximately 76% are assigned as choroidal or ciliary (Cancer Research UK; https://www.cancerresearchuk.org/health-professional/cancer-statistics/statistics-by-cancer-type/eye-cancer/incidence#heading-Four), a proxy for UM[76].
- Penetrance: 0.168
    - This is based on analysis of penetrance in first degree relatives of probands, for which we use a weighted combination of empirically derived loss-of-function and missense penetrances[26]
- Genetic heterogeneity: 0.02
    - This is based on sequencing of *BAP1* in a series of 432 unselected individuals with uveal melanomas in the Finnish population[77]
- Allelic heterogeneity: 1 (BA1), 0.1 (BS1), in accordance with existing ClinGen Variant Curation Expert Panel (VCEP) guidelines. We further created a BS1_sup threshold of 0.05, a more conservative approach than that deployed in recent *BRCA1/BRCA2* VCEP guidance for which a threshold of 0.02 was applied.

These parameters allowed generation of allele frequency thresholds for BA1, BS1 and BS1_sup of 4.56e-05, 4.56e-06 and 2.28e-06, respectively[20].

To construct the benignity truth set of missense variants, variants overlapping the coding regions of *BAP1* (ENST00000460680) were extracted from both gnomAD (v2.1.1 exomes) and UK Biobank. Allele counts in both datasets were tallied for each identified *BAP1* variant, both for individual non-founder ethnicities and for the total datasets. For each variant and AF threshold (BA1, BS1 and BS1_sup), we applied a population-specific allele count threshold for each ethnicity, which was calculated as the upper 95% of a Poisson distribution with k = the subpopulation size, and rate of occurrence = the AF threshold, as previously described[75]. Variants present at a count greater than the respective allele count threshold in at least one non-founder population were flagged as eligible for the respective evidence code. To

minimise the impact of stochastic variation in smaller subpopulations, we stipulated that a variant had to be observed at least twice in a given subpopulation to be eligible for benignity.

Variants were annotated with scores from meta-predictor REVEL[56], and awarded evidence points for benignity as per the ClinGen SVI-approved thresholds described by Pejaver *et al*.[78]

Variants were excluded from the benignity truthset if they had indicators of pathogenicity, namely REVEL scores > 0.644[78], SpliceAI scores > 0.2 or any existing pathogenic or likely pathogenic ClinVar annotation (≥2* review status).

Remaining variants were assigned to the benignity truth set if they were: (i) assigned a BA1 stand-alone population frequency flag, or (ii) assigned a BS1 or BS1_sup flag AND points for BP4, namely a REVEL score ≤ 0.290 (REVEL threshold for BP4_sup, as per Pejaver *et al*.[78]) – in accordance with ACMG combination rules.

Supplementary Method 14: UKBB PheWAS Analysis

The same strategies as described in a previous study were used to perform the PheWAS analysis[17]. Whole-exome sequencing (WES) data from 454,787 individuals in UK Biobank was used to identify *BAP1* variants in UKBB[27]. The WES data was stored as population-level VCF files that were aligned to GRCh38 and provided via the UKBB RAP (Research Access Platform). Several QC procedures were performed. Firstly, bcftools[79] was used to split multiallelic sites and to left correct and normalise indels. Then, variants that failed QC steps were removed from analyses. QC categories and values were as follows: 1) read depth < 7, 2) genotype quality <20, 3) the binomial test *p*-value for the reads of alternate allele versus the reads of reference allele ≤0.001 for heterozygous genotypes. For the indel genotypes, only variants with a read depth ≥10 and genotype quality ≥20 were kept. The variants that didn't pass the QC categories were set as null. After filtering, for a given variant, if more than 50% of its genotypes were missing, the variant was excluded from downstream analyses. VEP v102[53] was used to annotate all variants, with each variant assigned to a gene based on the primary MANE select v0.97[52] transcript with the most severe consequence. Variants found in UK Biobank were then annotated with SGE functional classification. In total, 57 SGE-depleted, 80 SGE-enriched and 754 SGE-unchanged variants with 297, 1,960 and 61,333 carriers respectively were identified. For the PheWAS analysis, only phenotypic consequences of variants classed as depleted by SGE functional classification were investigated. For the SGE-depleted *BAP1* variants, several rare variants burden test masks were created, including all *BAP1* SGE-depleted variants, *BAP1* HC PTVs predicted as depleted only, *BAP1* missense variants predicted as depleted only, *BAP1* HC PTVs plus missense variants and *BAP1* HC PTVs plus missense variants with the remaining PTV contained in UKBB. To make comparisons, masks including all HC PTVs in UKBB, missense variants with CADD scores > 25 and HC PTVs plus missense variants with CADD scores > 25 were also created. In addition, five *in silico* masks for EVE and REVEL scores were included, with higher score value cut-offs increasing the stringency for predicted pathogenicity with both tools: EVE≥0.5, EVE≥0.7, EVE≥0.75, REVEL≥0.5 and REVEL≥0.7. Cancer phenotypes were queried from cancer registry data. To evaluate the association between *BAP1* variants and overall cancer risk, the following phenotypic variables were generated: all cancer combined, all cancers combined excluding blood cancers, all cancers combined excluding skin cancers and all cancers combined

excluding blood and skin cancers. In addition to cancer phenotypes, the association between *BAP1* variants and a list of dichotomous and quantitative traits was also assessed (Supplementary Table 8). The regression models called from the 'statsmodels' package[80] implemented in python v3.7 with family set to 'binomial' and 'gaussian' for dichotomous and quantitative traits, respectively, were applied. For all regression models, age, age-squared, sex, WES sequencing batch, and the first ten genetic principal components described by Bycroft *et al*[81], were included. All the above-mentioned analyses were performed on UKBB RAP. All code and scripts can be found here: https://github.com/mrcepid-rap.

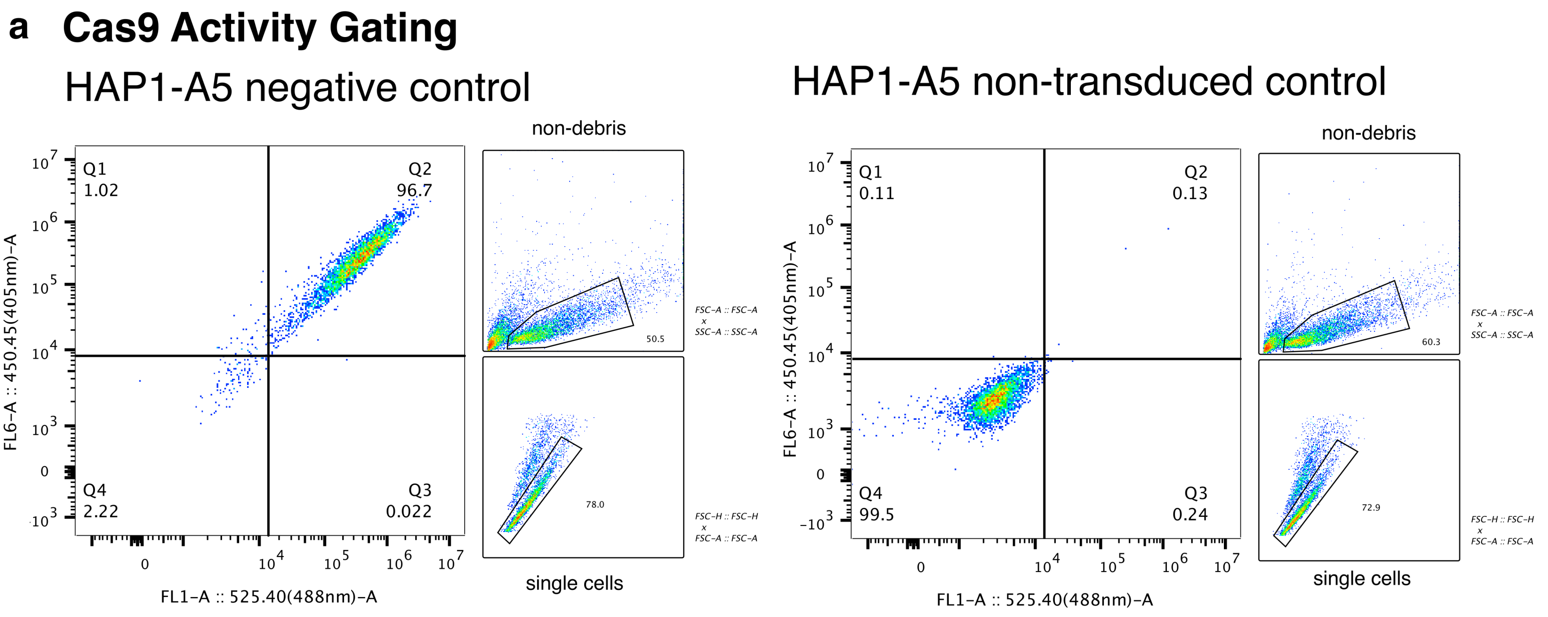

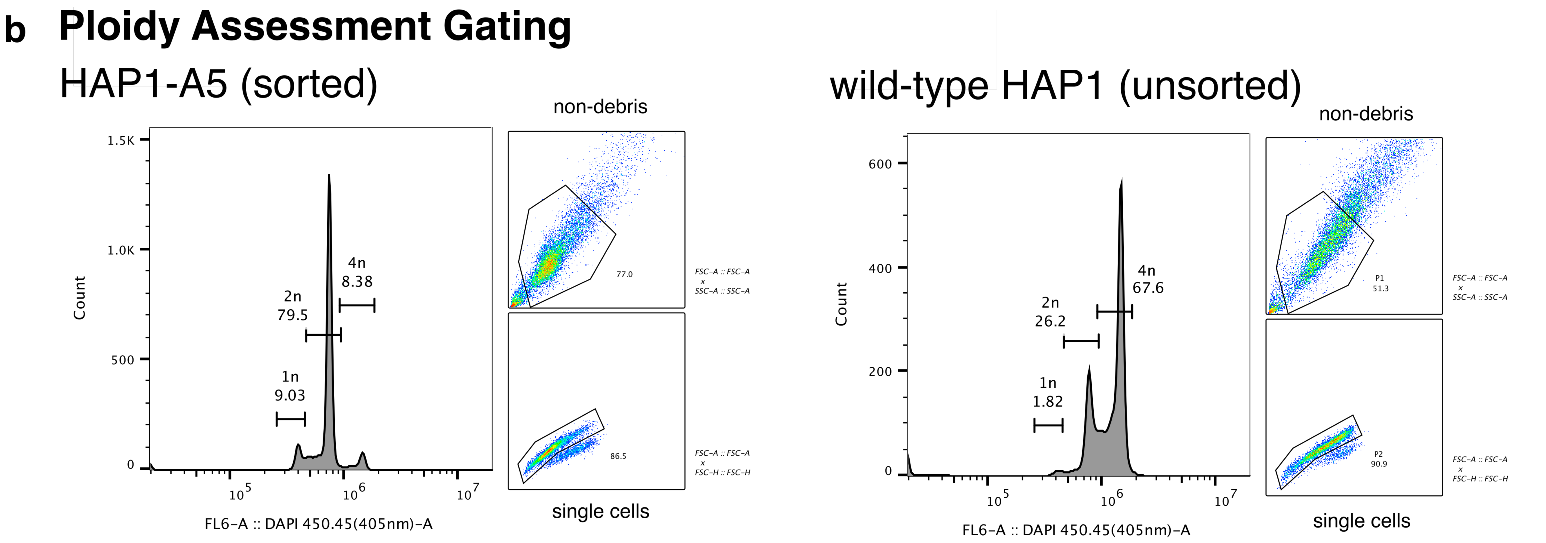

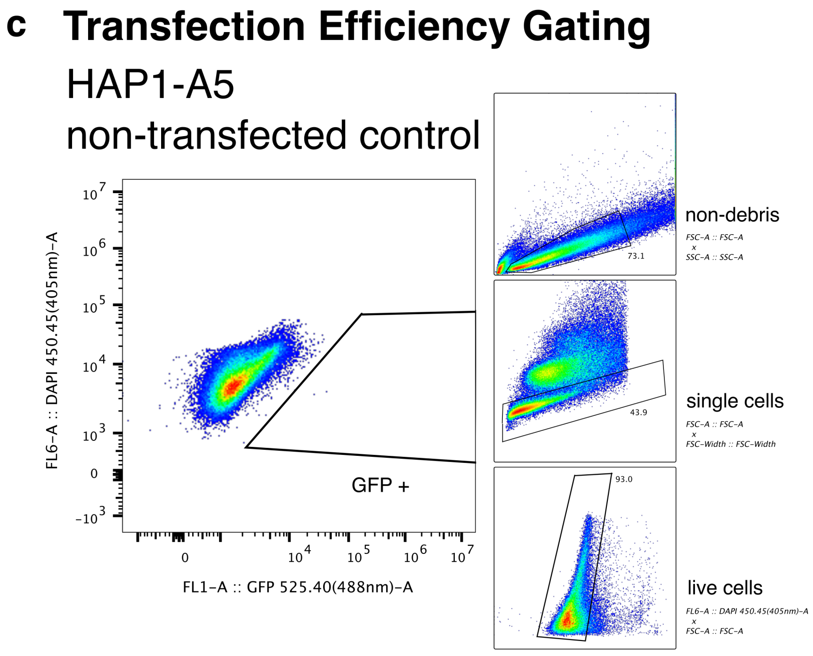

Supplementary Figure 1: Representative FACS gating. a. Cas9 activity assay gating on 10,000-20,000 cells at 72hrs post-transduction. Non-debris/cells were gated as a primary population using FSC-A and SSC-A using HAP1-A5 cells that were transduced with the negative control construct 'pKLV2-U6gRNA5(Empty)-PGKBFP2AGFP-W' (Addgene, 67979). Single cells were gated using FSC-H and FSC-A. This population was plotted for 405nMm-A (BFP) and 488nm-A (GFP) intensity and gated into quadrants (left panel). BFP- GFP- were distinguished in the lower left Q4 quadrant (right panel) using cells that were not transduced with the 'pKLV2-U6gRNA5(gGFP)-PGKBFP2AGFP-W' (Addgene, 67980) construct. Uniform gates were then applied to all sample populations. Blue-green-red shows increasing cell count. **b.** Ploidy assessment gating on 10,000-20,000 cells per sample. Non-debris/cells were gated as a primary population using FSC-A and SSC-A using HAP1-A5 clonal line bank cells. Single cells were gated using FSC-A and FSC-H. 1n and metaphase-arrested 2n (haploid) cells were distinguished as gates, high haploidy was expected as the clonal bank was sorted for haploidy (left panel). Metaphase arrested 4n (diploid) cells were distinguished in a gate on unsorted HAP1 cells which were expected to be moderately diploid (right panel). Uniform gates were then applied to all sample populations. Blue-green-red shows increasing cell count. **c.** Transfection efficiency assay gating on 50,000 cells. HAP1-A5 cells not transfected with 'pMax-GFP' (Lonza) were used for gating. Non-debris/cells were gated as a primary population using FSC-A and SSC-A. Single cells were gated using FSC-A and FSC-W. Live cells were then gated using DAPI (405nm)-A and FSC-A and a gate with an absence of GFP+ cells distinguished using DAPI (405nm)-A and GFP (488nm)-A. Uniform gates were then applied to all sample populations. Blue-green-red shows increasing cell count.

Supplementary Information References

67. Asada, S. *et al.* Mutant ASXL1 cooperates with BAP1 to promote myeloid leukaemogenesis. *Nat Commun* **9**, 2733 (2018).
68. Metzakopian, E. *et al.* Enhancing the genome editing toolbox: Genome wide CRISPR arrayed libraries. *Sci Rep* **7**, (2017).
69. Rice, P., Longden, I. & Bleasby, A. EMBOSS: The European Molecular Biology Open Software Suite. *Trends in Genetics* **16**, 276–277 (2000).
70. Needleman, S. B. & Wunsch, C. D. A general method applicable to the search for similarities in the amino acid sequence of two proteins. *J Mol Biol* **48**, 443–453 (1970).
71. DeWeirdt, P. C. *et al.* Genetic screens in isogenic mammalian cell lines without single cell cloning. *Nat Commun* **11**, (2020).
72. Gonçalves, E. *et al.* Minimal genome-wide human CRISPR-Cas9 library. *Genome Biol* **22**, (2021).
73. Li, W. *et al.* MAGeCK enables robust identification of essential genes from genome-scale CRISPR/Cas9 knockout screens. *Genome Biol* **15**, 554 (2014).
74. Clement, K. *et al.* CRISPResso2 provides accurate and rapid genome editing sequence analysis. *Nat Biotechnol* **37**, 224–226 (2019).
75. Whiffin, N. *et al.* Using high-resolution variant frequencies to empower clinical genome interpretation. *Genetics in Medicine* **19**, 1151–1158 (2017).
76. Kaliki, S. & Shields, C. L. Uveal melanoma: relatively rare but deadly cancer. *Eye* **31**, 241–257 (2017).
77. Repo, P. *et al.* Population-based analysis of BAP1 germline variations in patients with uveal melanoma. *Hum Mol Genet* **28**, 2415–2426 (2019).
78. Pejaver, V. *et al.* Calibration of computational tools for missense variant pathogenicity classification and ClinGen recommendations for PP3/BP4 criteria. *The American Journal of Human Genetics* **109**, 2163–2177 (2022).
79. Li, H. A statistical framework for SNP calling, mutation discovery, association mapping and population genetical parameter estimation from sequencing data. *Bioinformatics* **27**, 2987–2993 (2011).
80. Seabold, S. & Perktold, J. *Statsmodels: Econometric and Statistical Modeling with Python*. *PROC. OF THE 9th PYTHON IN SCIENCE CONF* http://statsmodels.sourceforge.net/ (2010).
81. Bycroft, C. *et al.* The UK Biobank resource with deep phenotyping and genomic data. *Nature* **562**, 203–209 (2018).
