# Supplementary material for: Saturation genome editing of BAP1 functionally classifies somatic and germline variants
Source: Nat Genet. 2024 Jul 5;56(7):1434–45. doi: 10.1038/s41588-024-01799-3 (PMC11250367; doi:10.1038/s41588-024-01799-3)
Supplement: Supplementary file 2 — Reporting Summary [file 41588_2024_1799_MOESM2_ESM.pdf]

nature portfolio

Corresponding author(s): David J. Adams, Andrew J. Waters

Last updated by author(s): Jan 24, 2024

Reporting Summary

Nature Portfolio wishes to improve the reproducibility of the work that we publish. This form provides structure for consistency and transparency in reporting. For further information on Nature Portfolio policies, see our Editorial Policies and the Editorial Policy Checklist.

Statistics

For all statistical analyses, confirm that the following items are present in the figure legend, table legend, main text, or Methods section.

n/a	Confirmed	
☐	☒	The exact sample size (*n*) for each experimental group/condition, given as a discrete number and unit of measurement
☐	☒	A statement on whether measurements were taken from distinct samples or whether the same sample was measured repeatedly
☐	☒	The statistical test(s) used AND whether they are one- or two-sided<br>*Only common tests should be described solely by name; describe more complex techniques in the Methods section.*
☐	☒	A description of all covariates tested
☐	☒	A description of any assumptions or corrections, such as tests of normality and adjustment for multiple comparisons
☐	☒	A full description of the statistical parameters including central tendency (e.g. means) or other basic estimates (e.g. regression coefficient) AND variation (e.g. standard deviation) or associated estimates of uncertainty (e.g. confidence intervals)
☐	☒	For null hypothesis testing, the test statistic (e.g. *F*, *t*, *r*) with confidence intervals, effect sizes, degrees of freedom and *P* value noted<br>*Give P values as exact values whenever suitable.*
☒	☐	For Bayesian analysis, information on the choice of priors and Markov chain Monte Carlo settings
☒	☐	For hierarchical and complex designs, identification of the appropriate level for tests and full reporting of outcomes
☐	☒	Estimates of effect sizes (e.g. Cohen's *d*, Pearson's *r*), indicating how they were calculated

Our web collection on statistics for biologists contains articles on many of the points above.

Software and code

Policy information about availability of computer code

Data collection	No software was used for data collection. Proprietary Illumina base-calling software/firmware on Miseq and HiSeq platforms was employed.
Data analysis	Please see 'Code Availability' section in the manuscript for GitHub and DOI for custom code used in the study.<br><br>Design and Analysis Software:<br><br>VaLiAnT version 1.0.0<br>FlowJo version 10.10<br>Geneious version 2023.0.4<br>R version 4.1.3 (2022-03-10) in RStudio Version 1.4.1106<br>QUANTS pipeline version 1.2.1.0<br>Nextflow version 22.04.3<br>Cutadapt version 3.2 with Python 3.8.6<br>FastQC version 0.11.9<br>SeqKit60 (stats) version 0.15.0<br>MultiQC61 version 1.10.1<br>pyCROQUET version 1.5.0

R Software versions:

FSA_0.9.4
pROC_1.18.4
ggpubr_0.6.0
DESeq2_1.34.0
DEGreport_1.30.3
ggridges_0.5.4
stringr_1.5.0
dplyr_1.1.2
tidyr_1.3.0
ggplot2_3.4.2
tidyverse_2.0.0

For manuscripts utilizing custom algorithms or software that are central to the research but not yet described in published literature, software must be made available to editors and reviewers. We strongly encourage code deposition in a community repository (e.g. GitHub). See the Nature Portfolio guidelines for submitting code & software for further information.

Data

Policy information about availability of data

All manuscripts must include a data availability statement. This statement should provide the following information, where applicable:

- Accession codes, unique identifiers, or web links for publicly available datasets
- A description of any restrictions on data availability
- For clinical datasets or third party data, please ensure that the statement adheres to our policy

BAP1 variant functional scores and classifications are freely available for all non-profit uses and are available here: https://github.com/team113sanger/Waters_BAP1_SGE and as Supplementary Data 1 and Supplementary Data 2.

FASTA and CRAM files generated in this study for HDR plasmid libraries and edited genomic DNA libraries are available through the European Nucleotide Archive (ENA) accession: 'PRJEB64778'

Raw counts generated through the QUANTS pipeline, and VaLiAnT and VEP annotation files,
are available for all non-profit uses through the BioStudies accession: 'S-BSST1222'

Mapped counts, experimental and bioinformatics methods are accessible through the MaveDB accession: 'urn:mavedb:00000662'.

GRCh38 used for all co-ordinates
gnomAD version 3
ClinVar downloaded 04/09/2023 (https://ftp.ncbi.nlm.nih.gov/pub/clinvar/vcf_GRCh38/archive_2.0/2023/) file= 'clinvar_20230903.vcf.gz'
ClinVar downloaded 20/09/2023 (https://ftp.ncbi.nlm.nih.gov/pub/clinvar/vcf_GRCh38/archive_2.0/2023/) file= 'clinvar_20230917.vcf.gz'

Research involving human participants, their data, or biological material

Policy information about studies with human participants or human data. See also policy information about sex, gender (identity/presentation), and sexual orientation and race, ethnicity and racism.

Reporting on sex and gender	na
Reporting on race, ethnicity, or other socially relevant groupings	na
Population characteristics	na
Recruitment	One patient is included in the study in a pedigree analysis. Permission was sought for inclusion, findings have been explained in person through genetic counseling and the patient has agreed to sign necessary declarations to allow for publication.
Ethics oversight	Department of Clinical Genetics, Leiden University Medical Center, Leiden, The Netherlands

Note that full information on the approval of the study protocol must also be provided in the manuscript.

Field-specific reporting

Please select the one below that is the best fit for your research. If you are not sure, read the appropriate sections before making your selection.

☒ Life sciences ☐ Behavioural & social sciences ☐ Ecological, evolutionary & environmental sciences

For a reference copy of the document with all sections, see nature.com/documents/nr-reporting-summary-flat.pdf

Life sciences study design

All studies must disclose on these points even when the disclosure is negative.

Sample size	No explicit sample size was decided upon a priori, rather we sought to assess all ~18,000 unique oligonucleotide species created through VaLiAnT, to comprehensively saturate all coding sequence and near-exon non-coding sequence of BAP1.<br><br>In order to maintain library complexity at the genome editing stage, 8 million cells were seeded 1 day before transfection. 6 million cells were sampled for timepoint-replicates gDNA extractions. 5 million cells were passaged at each timepoint to maintain culture through the screen.<br><br>Some variants were edited into the genome in multiple instances through over-lapping target regions and HDR repair libraries - subsequent to editing and during analysis steps, separate editing events were combined into a a single metric value through weighted mean calculations.
Data exclusions	Excluded time-point replicates and reasons for exclusion can be see in Supplementary Table 1 'analysis_status' column. Exclusion was either due to strong positional effect during editing or library indexing error.<br><br>Variants with fewer than 10 counts (generated through the QUANTS pipeline from CRAM file analysis) were excluded during analysis steps.
Replication	All transfections were performed in triplicate. Separate triplicates were maintained in culturing and sampling. When transfections failed, all three replicates failed, when transfections were successful all three replicates were successful. Successful transfection was determined by high cell survival post transfection and puromycin selection compared to non-transfected controls which were always included.<br><br>Data from 40/44 total HDR libraries were processed (library A and library B) at the analysis stage, with high reproducibility seen between Library A and Library B experiments (Fig.4a).
Randomization	Variants were edited into genomic loci in multiplex. ~1000 unique variants were integrated at each transfection, with variants related by proximity. gDNA libraries A and B for the same target regions were grouped into the same HiSeq sequencing run. All time point replicates for a target region were grouped together in the same HiSeq sequencing run. All tiled target regions (multiple target regions for larger exons) were grouped such that all gDNA libraries were grouped by exon in the same HiSeq sequencing run. Allocation of target regions to sequencing runs after these groupings were made was dictated by the order in which target regions were selected to be experimented upon (ie. when gDNA libraries were ready to be sequenced), which was essentially random. Groupings were as follows:<br><br>1 A+B, 3 A, 7 A+B, 9 A+B, 15 A+B<br>5 A+B, 10 A+B, 14 A+B<br>11.1 A+B, 11.2 A+B, 12.1 A+B, 12.2 A+B<br>2 A+B, 4 A+B, 17.1 A+B, 17.2 A+B<br>13.1 A+B, 13.2 A+B, 13.3 A+B<br>6 A+B, 8 B, 16 A+B<br><br>All plasmid HDR libraries were sequenced on the same MiSeq sequencing run.
Blinding	Functional scores and classification calculations were performed en masse, independently of known pathogenicty status. Assumptions were made about the likely minimal functional effect of synonymous and intronic variants in normalization processes, during which variants were systemically identified for inclusion using VEP. Blinding was not relevant to analyses or experiments in that all functional effect classifications and conclusions (including those for synonymous and intronic variants) emanated a posteriori after empirical data collection and analysis.

Reporting for specific materials, systems and methods

We require information from authors about some types of materials, experimental systems and methods used in many studies. Here, indicate whether each material, system or method listed is relevant to your study. If you are not sure if a list item applies to your research, read the appropriate section before selecting a response.

Materials & experimental systems

n/a	Involved in the study	
☐	☒	Antibodies
☐	☒	Eukaryotic cell lines
☒	☐	Palaeontology and archaeology
☒	☐	Animals and other organisms
☒	☐	Clinical data
☒	☐	Dual use research of concern
☒	☐	Plants

Methods

n/a	Involved in the study	
☒	☐	ChIP-seq
☐	☒	Flow cytometry
☒	☐	MRI-based neuroimaging

Antibodies

Antibodies used	SC-28383, Santa Cruz Biotechnology, Dallas, Texas, USA
Validation	The histological sections of human primary melanoma samples allow for internal validation as tumour cells (expected BAP1 negative)

Validation	and immune infiltrate (expected BAP1 positive) can be distinguished with 1:50 dilutions of primary antibody, seen in at least three replicates. Data sheet from Santa Cruz can be found here: https://datasheets.scbt.com/sc-28383.pdf

Eukaryotic cell lines

Policy information about cell lines and Sex and Gender in Research

Cell line source(s)	A HAP1 LIG4- cell line (HZGHC000759c005) with a 10bp deletion in LIG4, and its wild-type control were obtained from Horizon Discovery. This line was transduced with Cas9 lentivirus to create a polyclonal line from which single cell clones were derived to the create the HAP1 A5 cell line.
Authentication	Authenticated by karyotype: mFISH using 30 metaphase spreads. Sanger sequencing over LIG4 lesion to confirm LIG4-. Cas9 activity analysis and metaphase-arrest ploidy assessment.
Mycoplasma contamination	Cells were tested for Mycoplasma by commercial PCR and confirmed to be negative.
Commonly misidentified lines (See ICLAC register)	No commonly misidentified cell lines were used in the study.

Flow Cytometry

Plots

Confirm that:

☒ The axis labels state the marker and fluorochrome used (e.g. CD4-FITC).

☒ The axis scales are clearly visible. Include numbers along axes only for bottom left plot of group (a 'group' is an analysis of identical markers).

☒ All plots are contour plots with outliers or pseudocolor plots.

☒ A numerical value for number of cells or percentage (with statistics) is provided.

Methodology

Sample preparation	To assess the Cas9 activity: cells were transduced with a BFP/GFP activity construct ‘pKLV2-U6gRNA5(gGFP)-PGKBFP2AGFP-W’ (Addgene, 67980), a control construct was also used ‘pKLV2-U6gRNA5(Empty)-PGKBFP2AGFP-W’ (Addgene, 67979) with FACS analysis performed on 10,000-20,000 cells for each condition with 405nm and 488nm channels for BFP+ and GFP+, respectively.<br><br>To assess ploidy: Metaphase-arrested cells were used to accurately assess ploidy in the cell population. Day 3 and day 21 post-transfection cells were used (see Online Methods: ‘Tissue culture, cell transfection and sampling’ for transfection conditions). 5-8 Million cells were treated with 0.2nM of nocodazole (Sigma) for 14hrs at 37°C. Cells were dissociated and ethanol fixed.<br><br>To assess transfection efficiency: an empty vector of the sgRNA expression plasmid ‘pMin-U6-ccdb-hPGK-puro’ (5275bp) and a GFP-expressing plasmid ‘pMax-GFP’ (Lonza, 3486bp), were transfected into HAP1-A5 cells as described in Methods: ‘Tissue culture, cell transfection and sampling’. 7.5μg of pMin and 15μg of pMax-GFP were used for the transfection. Cells were dissociated at 3 days post-transfection. The live cells were incubated with 10μg/mL of DAPI for 30min at room temperature before proceeding to FACS analysis.
Instrument	LSRFortessaTM (BD Biosciences) FACS machine with low flow rate settings (ploidy and transfection)<br><br>CytoFLEX for Cas9 Activity
Software	FlowJo version 10.10
Cell population abundance	For ploidy and Cas9 activity: Analysis was performed on at least 1 x10^4 cells (selected in SSC-A vs FSC-A gate).<br>For transfection efficiency analysis was performed on at least 5 x10^4 cells (selected in SSC-A vs FSC-A gate).
Gating strategy	For Cas9 activity: gating for singlet cells with BFP (405nM) and GFP (488nM). Supplementary Fig. 1a.<br>For ploidy: gating for singlet cells with DAPI signal at 405nM channel. Supplementary Fig.1b<br>For transfection efficiency: GFP positive cells were determined by gating with 405nM (DAPI) and 488nM (GFP) channels. Supplementary Fig.1c.

☒ Tick this box to confirm that a figure exemplifying the gating strategy is provided in the Supplementary Information.
