# Supplementary figures and images for: Saturation genome editing of BAP1 functionally classifies somatic and germline variants
Source: Nat Genet. 2024 Jul 5;56(7):1434–45. doi: 10.1038/s41588-024-01799-3 (PMC11250367; doi:10.1038/s41588-024-01799-3)

Figure 6 b top panel

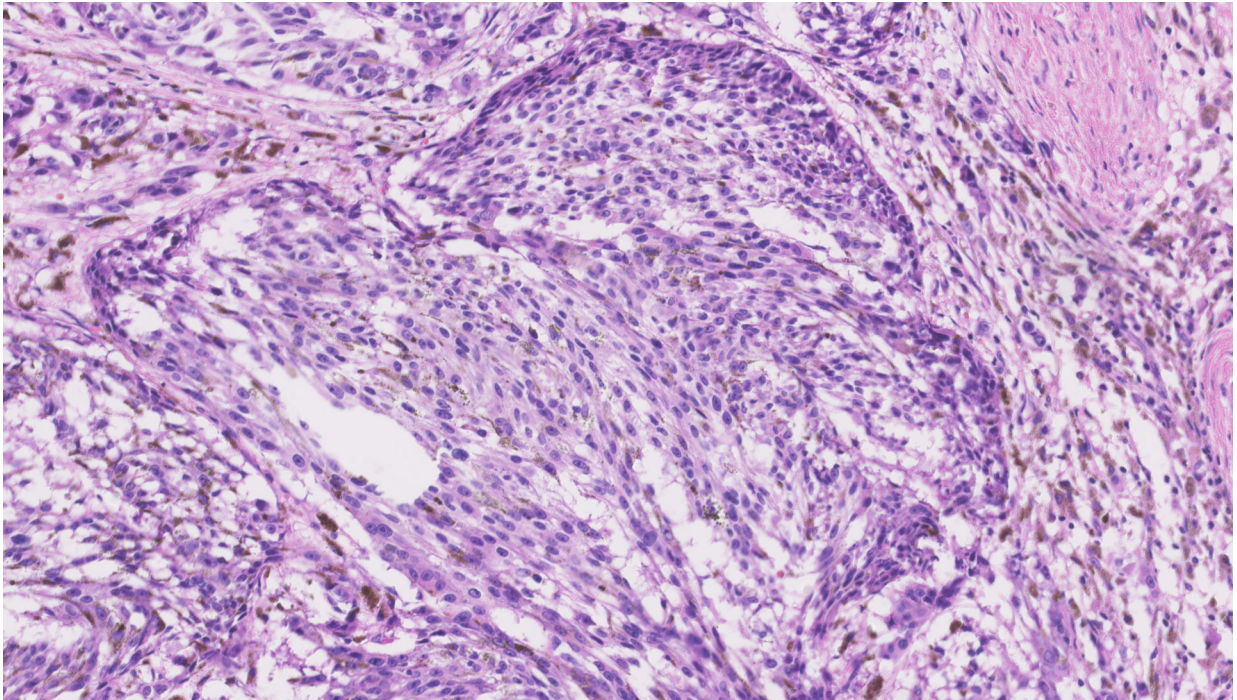

Figure 6 b bottom panel

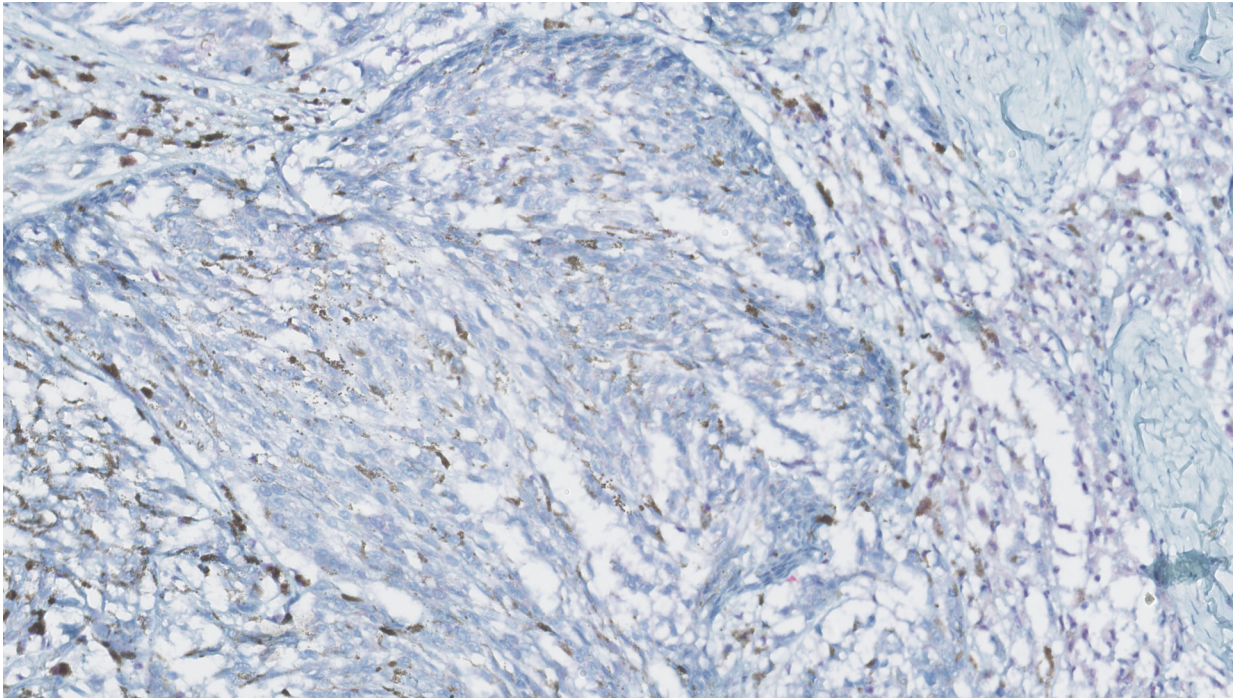

Supplement: Supplementary file 12 — Statistical and graph source data and micrographs. [file 41588_2024_1799_MOESM12_ESM.zip › source_data_figure_6/source_data_fig_6_b.pdf]
